# Preoperative Anemia Is a Predictor of Worse Postoperative Outcomes Following Open Pancreatoduodenectomy: A Propensity Score-Based Analysis

**DOI:** 10.3389/fmed.2022.818805

**Published:** 2022-05-13

**Authors:** Jing-Yong Xu, Xiao-Dong Tian, Yin-Mo Yang, Jing-Hai Song, Jun-Min Wei

**Affiliations:** ^1^Department of General Surgery, Department of Hepato-Bilio-Pancreatic Surgery, Beijing Hospital, National Center of Gerontology, Institute of Geriatric Medicine, Chinese Academy of Medical Sciences, Beijing, China; ^2^Department of General Surgery, Peking University First Hospital, Beijing, China

**Keywords:** anemia, complication, mortality, pancreatoduodenectomy, propensity score matching

## Abstract

**Background:**

Preoperative anemia is a common clinical situation proved to be associated with severe outcomes in major surgeries, but not in pancreatic surgery. We aim to study the impact of preoperative anemia on morbidity and mortality in patients undergoing open pancreatoduodenectomy and use propensity score matching (PSM) to balance the basal data and reduce bias.

**Methods:**

We analyzed the data of consecutive patients undergoing open pancreatoduodenectomy with a complete record of preoperative hemoglobin, at two pancreatic centers in China between 2015 and 2019. Anemia is defined as hemoglobin less than 12 g/dl for male and 11 g/dl for female, following Chinese criteria. We compared clinical and economic outcomes before and after PSM and used logistic regression analysis to assess the correlation between variables and anemia.

**Results:**

The unmatched initial cohort consisted of 517 patients. A total of 148 cases (28.6%) were diagnosed with anemia at admission, and no case received a preoperative blood transfusion or anti-anemia therapy. After PSM, there were 126 cases in each group. The rate of severe postoperative complications was significantly higher in the anemia group than in the normal group (43.7% vs. 27.0%, *p* = 0.006), among which the differences in prevalence of clinically relevant postoperative pancreatic fistula (CR-POPF) (31.0% vs. 15.9%, *p* = 0.005) and cardiac and cerebrovascular events (4.0% vs. 0.0%, *p* = 0.024) were the most significant. The costs involved were more in the anemia group (26958.2 ± 21671.9 vs. 20987.7 ± 10237.9 USD, *p* = 0.013). Among anemic patients, receiver operating characteristic (ROC) curve analysis shows the cut-off value of hemoglobin, below which, patients are prone to suffer from major complications (104.5 g/l in male and 90.5 g/l in female). Among all patients, multivariate analysis showed that preoperative obstructive jaundice [odds ratio (OR) = 1.813, 95% confidence interval (CI) (1.206–2.725), *p* = 0.004] and pancreatic ductal adenocarcinoma [OR = 1.861, 95% CI (1.178–2.939), *p* = 0.008] were predictors of anemia. Among paired patients, preoperative anemia [OR = 2.593, 95% CI (1.481–5.541), *p* = 0.001] and malignant pathology [OR = 4.266, 95% CI (1.597–11.395), *p* = 0.004] were predictors of postoperative severe complications.

**Conclusion:**

Preoperative anemia is a predictor of worse postoperative outcomes following open pancreatoduodenectomy and needs to be identified and treated.

## Introduction

Preoperative anemia is a common clinical situation, ranging from 25 to 40% in large observational studies (1, 2). Many studies have proved the association between preoperative anemia and postoperative mortality, morbidity, and prolonged length of hospital stay (3, 4). Moreover, preoperative anemia may also increase the rate of perioperative blood transfusion, which has been reported to be a risk factor for worse postoperative outcomes (5). Although there has been a long tradition that anemia can be corrected easily with transfusion, the treatment of preoperative anemia was still ignored and remained controversial to a certain extent (6).

Pancreatoduodenectomy (PD) is one of the major abdominal operations which is associated with high postoperative mortality and morbidity. There are many risk factors throughout the whole perioperative procedure (7). Although preoperative anemia has been proved to be a risk factor in many retrospective studies in cardiac and non-cardiac surgeries, the actual role that it plays in pancreatic surgery is still unclear. In this study, we aim to reveal the association between preoperative anemia and the adverse outcomes in open pancreatoduodenectomy by using propensity score matching (PSM) to balance the relative factors and reduce bias between anemia and non-anemia groups.

## Materials and Methods

### Patients and Baseline Characteristics

**FIGURE 1:**
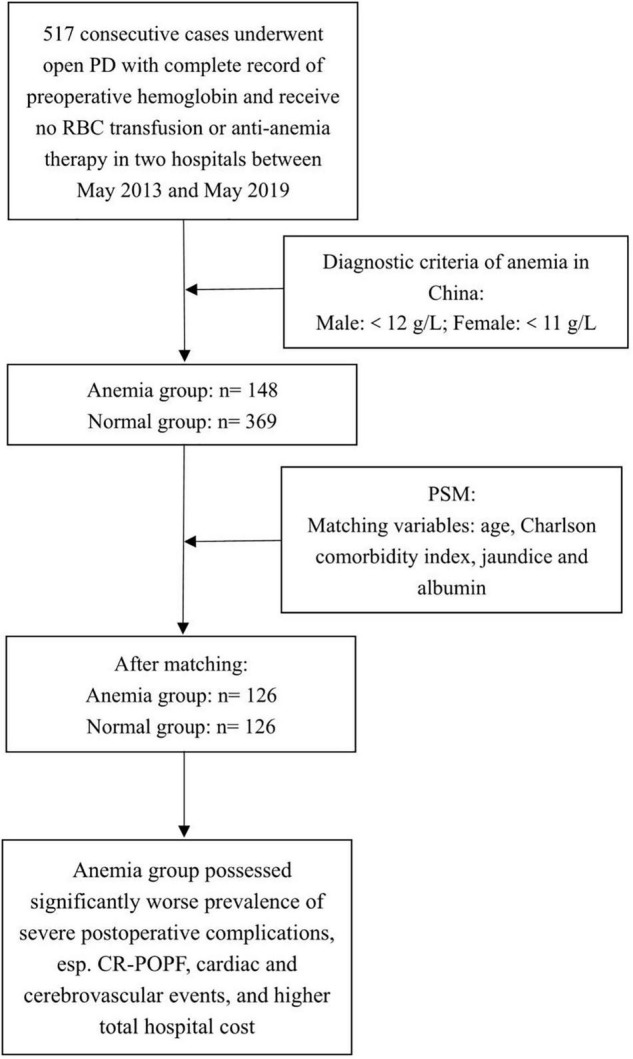
Flowchart of the study.

Data of consecutive patients with complete records of preoperative hemoglobin were analyzed retrospectively. All patients received open PD at two university hospitals in China between May 2015 and May 2019. Figure 1 shows the flowchart of this study. The local ethics committee approved the usage and publication of these data. Written informed consent was not considered necessary by the ethics committee because of the blinded data and retrospective design (Approval letter No. 2018BJYYEC-196-02).

Baseline characteristics included age, gender, body mass index (BMI), American Society of Anesthesiologists (ASA) classification, and preoperative obstructive jaundice. Age-adjusted Charlson comorbidity index (aCCI) was used to assess the comorbidities (8). In China, anemia was defined according to both the level of hemoglobin and gender. Hemoglobin less than 12 g/l in male and 11 g/l in female were defined as anemia (9). All recruited patients received no preoperative blood transfusion and supplement therapy.

Several nutritional variables such as albumin, nutritional risk, and malnutrition were included. The nutritional risk was defined by nutritional risk screening 2002 (NRS2002) (10) and malnutrition was defined by the global leadership initiative malnutrition (GLIM) diagnosis criteria (11).

### Intraoperative and Postoperative Data

The operation method of open PD was unified in our two institutes due to long-term cooperation (7). We performed the total mesopancreatic resection (TMpE) and standard lymph node dissection for malignance and the traditional procedure for benign diseases. Some operative data that might affect outcomes, especially complications, were compared, including pancreatic duct diameter, vein reconstruction, and way of pancreatic anastomosis. Intraoperative data included the duration of the procedure, volumes of blood loss, intraoperative red blood cells, and fluid infusion. Because this is a study focusing on the procedure, not tumor treatment and survival, we recorded the pathology in two parts: malignant and benign. The malignant diseases consisted of pancreatic ductal adenocarcinoma (PDAC), Vater’s ampullary carcinoma, duodenal carcinoma, and common bile duct carcinoma. The benign diseases were divided into two parts: chronic pancreatitis and benign tumors, which contained adenoma of the duodenum and bile duct, pancreatic neuroendocrine tumors, and benign cystic lesions. Since there were few patients in each diagnosis, we analyzed them together. Also, we recorded PDAC separately because it possessed a higher malignance grade and tighter relationship with anemia, and only resectable PDAC was recruited in this study.

All patients received similar and standard treatment after the operation in our two hospitals. Parenteral nutrition support started on the second postoperative day. Enteral nutrition or oral intake began on the third day, and we gradually increased the amount. The goal of daily energy was 25 kcal/kg, and the goal of protein was 1.2–1.5 g/kg per day. Albumin was administrated routinely for 3 days after the operation, and we decided whether to continue after a reassessment of the concentration. Prophylactic antibiotics and somatostatin were also used.

Complications were recorded according to the Claviene-Dindo (CD) classification system (Minor: I-II; Major: III-IV) and the total number of death (grade V in the CD system) was recorded separately (12). We defined that all the studied postoperative outcomes happened during the hospital stay. Postoperative pancreatic fistula (POPF) was defined and graded according to the 2016 International Study Group of Pancreatic Surgery (ISGPS) classification and clinically relevant POPF (CR-POPF) contained both grades B and C (13). Non-fistulous complications like postpancreatectomy hemorrhage (PPH), delayed gastric emptying (DGE), biliary fistula, abdominal infection, and cardiac and cerebrovascular events were also included, and ISGPS definitions and classifications of PPH and DGE were followed (14, 15).

In-hospital reoperation rate, postoperative length of stay (LOS), 30-day readmission rate, perioperative mortality, and total hospital costs were recorded. Total hospital costs only contained the direct cost on the hospitalization bill including fees for operation, drugs and medical equipment, nursing care, and other medical services, such as consultation.

### Propensity Score Matching

Propensity score matching was applied to achieve a balance between the two groups. We selected those variables that were significantly different between the two groups in the original data analysis by groups comparison and logistic analysis, including age, albumin, CCI, and preoperative obstructive jaundice to generate the propensity score, and binary logistic regression with selected variables was used to generate continuous propensity scores from 0 to 1. Patients were matched by a matching ratio of 1:1 based on the propensity score with a standard caliper width of 0.02 (16).

### Statistical Analysis

The data were collected and checked by two staff to ensure accuracy at the two institutions. IBM SPSS Statistics (Ver. 26.0, IBM Corp., Armonk, NY, United States) was used by professional statisticians to do the statistical analysis. Categorical data were analyzed using the chi-square test or Fisher exact test. Continuous data were tested by Student’s unpaired *t*-test. CCI was shown by median and interquartile range (IQR) and analyzed by the Mann-Whitney U test. The cut-off values of hemoglobin were calculated by maximizing the sensitivity and specificity using the Youden index, and the areas under the receiver operating characteristic (ROC) curves were compared. Multivariable logistic regression analysis was used to evaluate the relationship between risk factors and anemia and postoperative severe complications, respectively, which was expressed as an odds ratio (OR) with 95% confidence intervals (CI). We determined the risk factors by referring to several published articles and what we had in our database, including age, sex, comorbidities, nutrition-related variables, pathology, and some intraoperative items (7, 17). We did the logistic analysis of the risk factors of anemia in the total cohort in order to reduce the error caused by missing cases, and we did the analysis of the risk factors of complications in the paired cohort in order to prevent the influence of bias. A value of *p* less than 0.05 was considered statistically significant.

## Results

### Basal Data of All Patients

In total, 517 consecutive patients were included and the main age was 62.0 ± 11.6 years (range: 16–88 years). The male: female ratio was 1.5:1 (307:210). A total of 148 cases (28.6%) were diagnosed with anemia at admission, and no case received a preoperative blood transfusion or anti-anemia therapy. A total of 151 (29.2%) cases had a history of long-term drinking, and 171 (33.1%) were heavy smokers. The median and IQR of aCCI were 4.0 (3.0, 5.0). A total of 301 (58.2%) patients had obstructive jaundice. Referring to nutrition status, the main BMI was 23.5 ± 3.3 kg/m^2^. The main plasma albumin concentration was 38.5 ± 6.3 g/l. A total of 319 (61.7%) cases were at nutritional risk by NRS2002, while 132 (25.5%) cases were malnourished, as determined by GLIM. According to the postoperative pathologic results, the malignance accounted for 77.6% and 151 (29.2%) cases had pancreatic ductal adenocarcinoma.

### Logistic Analysis of Risk Factors of Preoperative Anemia

**FIGURE 2:**
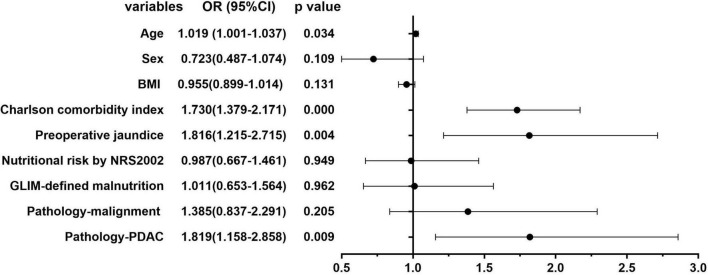
Univariate analysis of the risk factors of preoperative anemia in all patients.

**FIGURE 3:**
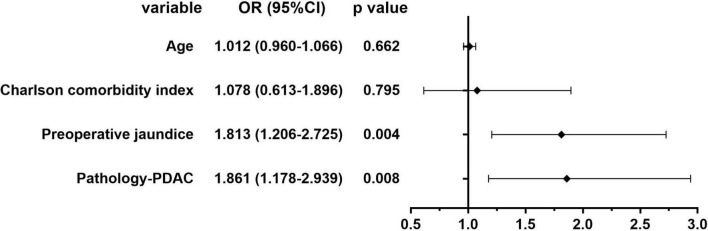
Multivariate analysis of the risk factors of preoperative anemia in all patients.

Figures 2, 3 show the results of univariate and multivariate analysis in all patients. We aimed to find out the risk factor of preoperative anemia. After multivariate analysis, preoperative obstructive jaundice [OR = 1.813, 95% CI (1.206–2.725), *p* = 0.004] and pathologic diagnosis of pancreatic ductal adenocarcinoma [OR = 1.861, 95% CI (1.178–2.939), *p* = 0.008] were proved to be two risk factors of preoperative anemia with statistical significance.

### Basal Data Comparison Before and After PSM

Table 1 shows the comparison of basal data between anemia and normal groups before and after PSM. Before PSM, we could see that patients who were older (63.7 vs. 61.8 years, *p* = 0.033) or with a more severe comorbidity assessed by aCCI [median 4.0 (IQR 1.0) vs. 4.0 (IQR 2.0), *p* = 0.013], especially preoperative obstructive jaundice (68.2% vs. 54.2%, *p* = 0.003), were more likely to develop anemia. And lower albumin (mean 35.3 ± 5.9 vs. 39.8 ± 6.0, *p* = 0.000) might be due to malnourishment or chronic consumption caused by original disease. After PSM, all baseline variables were balanced.

**TABLE 1:** Basal data comparison between anemia and no anemia groups.

Variable	Before PSM		*P*	After PSM		*P*
			
	Anemia group (*n* = 148)	Non-anemia group (*n* = 369)		Anemia group (*n* = 126)	Non-anemia group (*n* = 126)	
Age, mean (SD), y	63.7 (10.4)	61.8 (11.9)	0.033	63.0 (10.5)	63.8 (10.2)	0.555
Sex, male, n (%)	96 (64.9)	211 (57.2)	0.108	51 (40.5)	41 (32.5)	0.191
ASA (I/II/III), n (%)	8/111/29 (5.4/75.0/19.6)	34/292/43 (9.2/79.1/11.7)	0.074	7/94/25 (5.5/74.6/19.8)	10/98/18 (7.9/77.8/14.3)	0.371
Charlson comorbidity index, median (IQR)	4.0 (1.0)	4.0 (2.0)	0.013	2.0 (2.0)	4.0 (1.0)	0.776
Preoperative obstructive jaundice, n (%)	101 (68.2)	200 (54.2)	0.003	83 (65.9)	89 (70.6)	0.417
**Nutrition status**						
BMI, mean (SD), kg/m^2^	23.2 (3.8)	23.7 (3.0)	0.131	23.2 (3.9)	23.9 (3.2)	0.146
Albumin, mean (SD), g/L	35.3 (5.9)	39.8 (6.0)	0.000	36.5 (5.5)	36.7 (5.8)	0.693
NRS2002-define nutritional risk	91 (61.5)	228 (61.8)	0.949	75 (59.5)	76 (60.3)	0.898
GLIM-defined malnutrition	38 (25.7)	94 (25.5)	0.962	29 (23.0)	34 (27.0)	0.467
**Operative data**						
Vein construction, n (%)	12 (8.1)	28 (7.6)	0.583	12 (9.5)	11 (8.7)	0.906
Duct-to-mucosa anastomosis, n (%)	75 (50.7)	200 (54.2)	0.487	66 (52.4)	63 (50.0)	0.752
Diameter of pancreatic duct, mm	2.91 ± 1.68	3.29 ± 1.89	0.307	2.77 ± 1.83	3.02 ± 1.90	0.297
Pathology, n (%)			0.091			0.256
PDAC	34 (23.0)	117 (31.7)		25 (19.8)	37 (29.4)	
Vater’s ampulla carcinoma	28 (18.9)	47 (12.7)		20 (15.9)	25 (19.8)	
Common bile duct carcinoma	40 (27.0)	100 (27.1)		34 (27.0)	29 (31.0)	
Duodenal carcinoma	25 (16.9)	40 (10.8)		25 (19.8)	21 (16.7)	
Chronic pancreatitis	4 (2.7)	15 (4.1)		4 (3.2)	1 (0.8)	
Benign tumors	17 (11.5)	50 (13.6)		13 (10.3)	8 (6.3)	

*ASA, American Society of Anesthesia; BMI, body mass index; NRS, nutritional risk screening; GLIM, global leadership initiative malnutrition; SD, standard deviation; IQR, interquartile range; PDAC, pancreatic ductal adenocarcinoma.*

### Outcomes Comparison Before and After PSM

Table 2 shows the comparison of outcome parameters between anemia and normal groups before and after PSM. For intraoperative data, blood loss and RBC transfusion were reported to be the risk factors for poor outcomes (5) and might be the bias in this study. After matching, the differences were balanced and the bias declined. Referring to postoperative data, we could see that the differences in several variables remained statistically obvious after PSM, including the rate of severe postoperative complications (43.7% vs. 27.0%, *p* = 0.006), especially the rate of CR-POPF and PPH, and the difference in total hospital costs. The difference in the prevalence of cardiac and cerebrovascular complications between the two groups became significant after matching (4.0% vs. 0.0%, *p* = 0.024).

**TABLE 2:** Complication and outcome comparison between anemia and no anemia groups.

Variable	Before PSM		*P*	After PSM		*P*
			
	Anemia group (*n* = 148)	Non-anemia group (*n* = 369)		Anemia group (*n* = 126)	Non-anemia group (*n* = 126)	
**Intraoperative**						
Operation duration, mean (SD), min	404.2 (134.6)	360.0 (108.8)	0.001	399.1 (128.1)	368.6 (111.8)	0.045
Blood loss, median (IQR), ml	500.0 (600.0)	400.0 (400.0)	0.000	500.0 (600.0)	500.0 (800.0)	0.655
Fluid infusion, mean (SD), ml	4320.0 (2443.2)	3937.4 (2458.2)	0.111	4272.9 (2454.0)	3944.1 (1529.3)	0.203
RBC transfusion, n (%)	86 (58.1)	118 (32.0)	0.000	72 (57.1)	77 (61.1)	0.522
RBC transfusion volume, median (IQR), ml	340.0 (680.0)	0.0 (340.0)	0.000	340.0 (600.0)	340.0 (680.0)	0.203
**Postoperative**						
CD III-V complications, n (%)	65 (43.9)	111 (30.1)	0.003	55 (43.7)	34 (27.0)	0.006
CR-POPF, n (%)	45 (30.4)	60 (16.3)	0.000	39 (31.0)	20 (15.9)	0.005
PPH Grade B/C, n (%)	27 (18.2)	40 (10.8)	0.023	25 (19.8)	16 (12.7)	0.125
Biliary fistula, n (%)	18 (12.2)	29 (7.9)	0.124	14 (11.1)	10 (7.9)	0.391
Abdominal infection, n (%)	26 (17.6)	30 (8.1)	0.002	21 (16.7)	11 (8.7)	0.058
DGE Grade B/C, n (%)	25 (16.9)	53 (14.4)	0.468	22 (17.5)	19 (15.1)	0.609
Cardiac and cerebrovascular events, n (%)	5 (3.4)	4 (1.1)	0.071	5 (4.0)	0 (0.0)	0.024
Reoperation, n (%)	9 (6.1)	17 (4.6)	0.488	8 (6.3)	6 (4.8)	0.582
Outcomes						
Postoperative LOS, mean (SD), day	27.3 (18.3)	25.6 (18.2)	0.324	27.0 (18.2)	24.9 (17.3)	0.346
30-day readmission rate, n (%)	3 (2.0)	5 (1.4)	0.576	3 (2.4)	2 (1.6)	0.651
Perioperative mortality, n (%)	8 (5.4)	7 (1.9)	0.042	7 (5.6)	1 (0.8)	0.066
Total hospital costs, mean (SD), USD	27116.8 (20853.6)	20335.9 (10460.9)	0.000	26958.2 (21671.9)	20987.7 (10237.9)	0.013

*RBC, red blood cell; CD, Claviene-Dindo; CR-POPF, clinically relevant -postoperative pancreatic fistula; PPH, Postopancreatectomy hemorrhage; DGE, Delayed gastric emptying; LOS, length of stay; SD, standard deviation; USD, United States dollar.*

### Estimation of the Reference Values of Hemoglobin

**FIGURE 4:**
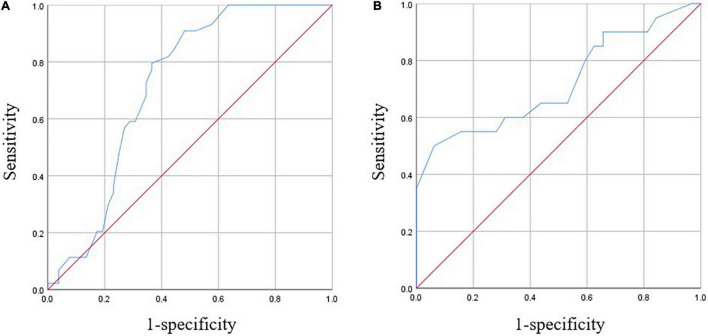
Receiver operating characteristic (ROC) curves of hemoglobin for major complications. **(A)** For males. **(B)** For females.

The results of the ROC analysis are displayed in Figure 4 and Table 3. Hemoglobin of 104.5 g/l in male patients and 90.5 g/l in female patients were proved to be the optimal cut-off values for predicting major complications with statistical significance.

**TABLE 3:** Estimation of the cut-off values of hemoglobin.

	Male (*n* = 96)	Female (*n* = 52)
Major complication, n (%)	44 (45.8)	20 (38.5)
Estimated cut-off value in Hgb, g/L	104.5	90.5
Area under the ROC curve	0.717, 95%CI (0.612,0.821)	0.720, 95%CI (0.568,0.873)
sensitivity	0.795	0.500
specificity	0.635	0.937
Youden Index	0.430	0.438
*p*-value	0.000	0.008

*Hgb, hemoglobin; ROC, receiver operating characteristic.*

### Logistic Analysis of Risk Factors of Postoperative Severe Complications

**FIGURE 5:**
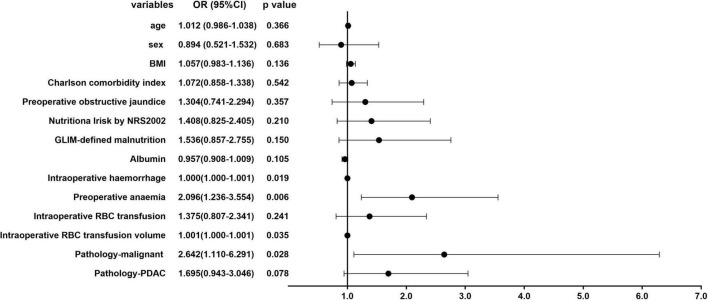
Univariate analysis of the risk factors of severe postoperative complications in patients after propensity score matching (PSM).

**FIGURE 6:**
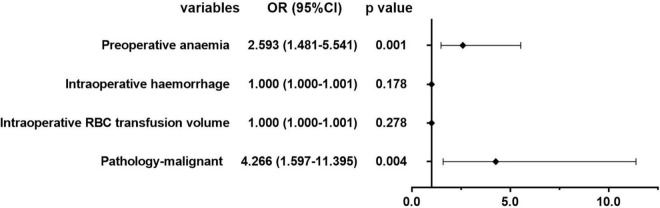
Multivariate analysis of the risk factors of severe postoperative complications in patients after PSM.

Figures 5, 6 show the univariate and multivariate analysis of the risk factors of severe postoperative complications in patients after PSM. Preoperative anemia [OR = 2.593, 95% CI (1.481–5.541), *p* = 0.001] and malignant pathology [OR = 4.266, 95% CI (1.597–11.395), *p* = 0.004] were proved to be predictors of postoperative severe complications.

## Discussion

Pancreatoduodenectomy is one of the major abdominal operations associated with high postoperative mortality and morbidity. There are many risk factors throughout the whole perioperative procedure, which may lead to systematic inflammation, metabolic and nutritional disorders including anemia (18). Preoperative anemia in pancreatic surgery was a prevalent condition accounting for 28.6% in this study, which is similar to the prevalence from other large observational studies in the field of major abdominal surgery. Many factors may result in preoperative anemia such as age, gender, comorbidities, and pathology (19). All these factors would affect the nutrition status because of a combination of malnourishment, malabsorption, chronic gastrointestinal hemorrhage, and consumption caused by malignant lesions, especially in the elderly (20, 21). So, nutrition-related types of anemia like iron deficiency anemia were proved to be the most common in surgical patients (2, 22). In this study, we found almost the same risk factors before PSM. Patients with anemia were older, with higher CCI and a higher prevalence of obstructive jaundice. Meanwhile, patients suffering from PDAC were prone to anemia.

We also found that anemic patients had lower albumin, but we treated it as a co-existing disorder caused by pancreatic diseases and nutritional changes. So, we did not put it into the logistic analysis. Not like albumin, traditional nutrition screening and assessment tools were not sensitive enough to indicate the existence of anemia in this study, maybe because the items in these two tools contained only phenotype and etiologic parameters but not the items reflecting the internal environment. Recently, the American Society for Enhanced Recovery and Perioperative Quality Initiative Joint developed a new tool named perioperative nutrition screen (PONS), which was based on a patient’s BMI, recent changes in weight, the recent decrease in dietary intake, and preoperative albumin level (23). There is no relative data on PONS in our database and we hope this new tool could be validated soon, and we need more studies and high-quality evidence to determine whether anemia can be a part of nutrition assessment.

Blood transfusion was thought to be a double-edged sword for pancreatic surgeons, which might cure anemia but also bring poor survival in patients with periampullary cancer (24). Meanwhile, preoperative anemia may increase the rate of perioperative blood transfusion (5). In this study, we found that both the proportion of intraoperative transfusion and the transfusion volume was significantly higher in the anemia group before PSM. On the other hand, blood transfusion and preoperative anemia were co-existing risk factors, but there is no agreement on the relative contribution of each of them, so they may become bias toward each other when we do the logistic analysis (25, 26). So, after PSM, we could see from Table 2 that the RBC transfusion rate and volume were balanced between the two groups, which means we reduced the bias to the utmost in order to make our results reliable.

Referring to outcomes, two aspects were mentioned in this study: intraoperative and postoperative. For intraoperative variables, the patients in the anemia group had longer operation time and more blood loss, which was the same as the results of a recent study (27). Blood loss was balanced after PSM to reduce the interaction with anemia and transfusion. For postoperative outcomes, many studies have proved the association between preoperative anemia and postoperative mortality, morbidity, and prolonged length of hospital stay in the field of elective major surgery, but the actual role that it plays in pancreatic surgery is still unclear. In our study, we found that the prevalence of severe postoperative complications was higher in the anemia groups after PSM, especially CR-POPFs and cardiac and cerebrovascular events. Anemia may lead to changes in blood composition and result in pathophysiologic changes which influence circulation. In a recent retrospective study, the authors found that preoperative anemia was independently associated with myocardial injury after non-cardiac surgery (28), and in some studies in cardiac surgery, the association between preoperative anemia and postoperative stroke was reported (29).

Since preoperative anemia is associated with increased postoperative complications and poor outcomes in patient after surgery, more surgeons agreed that intervention should be incorporated into routine care before a major operation (30). However, how to treat preoperative anemia was still controversial to a certain extent (6). International guidelines support the use of intravenous iron to correct anemia in patients before surgery, but a recent randomized control trial (RCT) showed no benefit from giving intravenous iron before the operation (31). So, more studies are needed to make an appropriate strategy toward preoperative anemia.

There are several limitations that may impact the analysis. First, this is a retrospective study and the sample size is relatively small. Second, data were limited to the immediate postoperative period and cannot reflect the influence on long-term outcomes, especially survival.

## Conclusion

The prevalence of preoperative anemia is high in pancreatic surgery. It is a predictor of worse postoperative outcomes following open pancreatoduodenectomy, such as severe postoperative complications, cardiac and cerebrovascular events, and higher hospital costs. It needs to be identified timely and treated before surgery, and more high-grade evidence is needed in the future.

## Data Availability Statement

The raw data supporting the conclusions of this article will be made available by the authors, without undue reservation.

## Ethics Statement

The studies involving human participants were reviewed and approved by Ethics Committee of Beijing Hospital. The ethics committee waived the requirement of written informed consent for participation.

## Author Contributions

J-HS, J-MW, Y-MY, and J-YX: conception and design. Y-MY and J-HS: administrative support. J-YX and X-DT: provision of study materials or patients, collection, assembly of data, data analysis, and interpretation. All authors wrote the manuscript and approved the final manuscript.

## Conflict of Interest

The authors declare that the research was conducted in the absence of any commercial or financial relationships that could be construed as a potential conflict of interest.

## Publisher’s Note

All claims expressed in this article are solely those of the authors and do not necessarily represent those of their affiliated organizations, or those of the publisher, the editors and the reviewers. Any product that may be evaluated in this article, or claim that may be made by its manufacturer, is not guaranteed or endorsed by the publisher.

## Abbreviations

PSM: propensity score matching
BMI: body mass index
ASA: American Society of Anesthesiologists
aCCI: Age-adjusted Charlson comorbidity index
NRS: nutritional risk screening
GLIM: global leadership initiative malnutrition
CD: Claviene-Dindo
POPF: postoperative pancreatic fistula
CR-POPF: clinically relevant postoperative pancreatic fistula
PPH: postpancreatectomy hemorrhage
DGE: delayed gastric emptying
ISGPS: International Study Group of Pancreatic Surgery
LOS: length of stay
IQR: interquartile range
OR: odds ratio
PONS: perioperative nutrition screen
SD: standard deviation
USD: United States dollar
RBC: red blood cell
RCT: randomized control trial
PDAC: pancreatic ductal adenocarcinoma.
